# Comparative gene expression supports the origin of the incisor and molar process from a single endite in the mandible of the red flour beetle *Tribolium castaneum*

**DOI:** 10.1186/2041-9139-4-1

**Published:** 2013-01-02

**Authors:** Joshua F Coulcher, Maximilian J Telford

**Affiliations:** 1 Department of Genetics, Environment and Evolution, University College London, Darwin Building, Gower Street, London, WC1E 6BT, UK

## Abstract

**Background:**

The biting edge of the primitive arthropod mandible consists of a biting incisor process and a crushing molar process. These structures are thought to be derived from a structure known as an endite but the precise details of this are not understood. Various hypotheses concerning the number of endites present in the arthropod mandible have been proposed.

In the developing embryo, the mandible has an inner and outer lobe that are likely to develop into the incisor and molar processes of the larval mandible; these two lobes are commonly held to be derived from separate endites and to be serially homologous to the galea and lacinia endites of the maxillary appendage respectively (Machida).

**Results:**

We undertook a study of the development of the embryonic mandible of the beetle *Tribolium castaneum* using the expression of developmental genes as markers of the developing endites in the mandible and maxilla.

The *Tribolium* ortholog of *paired* (*Tc-prd*) has expression domains in the developing maxillary and labial endites as well as the inner and outer lobes of the mandible. Following the expression of *Tc-prd* in the developing mandible through to late stage embryos shows that the molar and incisor process develop from the inner and outer lobes respectively.

In addition to *Tc-prd, w*e compared the expression of genes in the endites of the maxilla to the mandible to draw conclusions about the number of endites in the mandible. Homologs of *dachshund* are typically expressed in the endites of mandibulate gnathal appendages*.* Comparison of the expression of *Tc-prd, Tribolium dachshund (Tc-dac)* and *Tribolium wingless* (*Tc-wg)* between the endites of the maxilla and the mandible suggest that, while there are two endites in the maxilla only a single endite is present in the mandible.

**Conclusions:**

Comparative gene expression suggests that the *Tribolium* mandible has a single endite from which both mandible lobes are derived. Our results do not support Machida’s hypothesis homologising the incisor and molar processes of the mandible to the galea and lacinia endites of the maxilla. We propose, instead, that both incisor and molar processes are derived from a single endite serially homologous to the lacinia of the maxilla.

## Introduction

**Figure 1:**
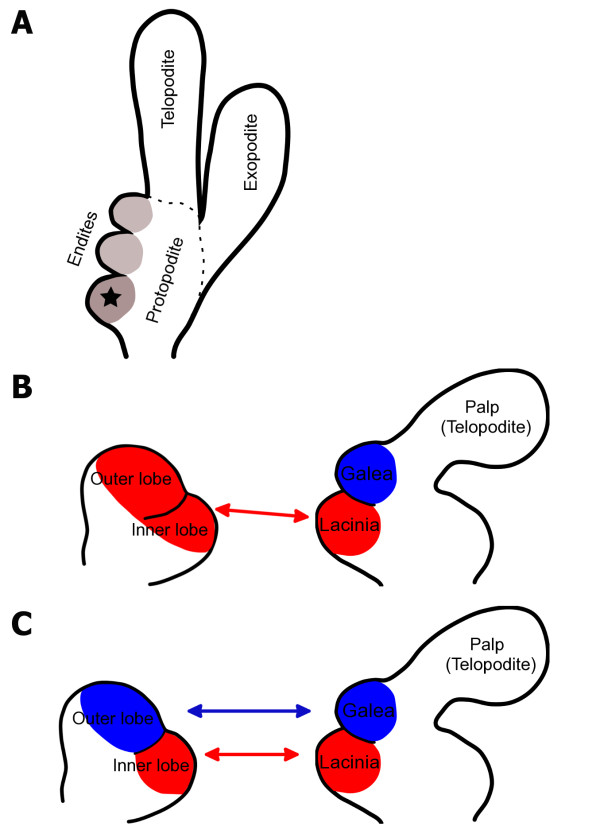
**Hypotheses of the serial homology of the mandibular inner and outer lobes to the maxillary lacinia and galea endites.** (**A**) Representation of a primitive embryonic biramous limb from which all post-antennal limbs have evolved. The limb consists of two branches (rami), the telopodite and exopodite which are attached to a proximal protopodite. There are multiple endites on the proximal-medial part of the limb. The proximal endite is indicated with a star. (**B**) A representation of the embryonic mandible with the inner and outer lobes of the mandible derived from one endite (shown in red). If the molar and incisor processes are derived from one endite, then they can only be serially homologous to one of the maxillary endites (such as the lacinia). (**C**) The inner (red) and outer (blue) lobes of the mandibular embryonic appendage have been hypothesised to be serially homologous to the lacinia (red) and galea (blue) of the maxilla by Machida. This hypothesis assumes that the mandible has two endites which develop into the molar and incisor processes.

The typical insect mandible is an unsegmented appendage with a biting edge consisting of an incisor and a molar process. Like all post-antennal appendages, it has evolved from a biramous limb consisting of two branches, a telopodite and an exopodite that are attached to the protopodite (the base) [1]. On the protopodite of diverse arthropod appendages, lobed structures called endites are found that are often involved in food processing (see Figure 1A).

Insects have lost the endites on the leg appendages but typically retain them on the mandibular, maxillary and labial appendages, otherwise known as the gnathal appendages. The canonical insect maxilla and labium both have two endites - the lacinia and galea on the maxilla and the postmentum and prementum on the labium.

Although endites typically consist of a lobe covered in bristle, there is a diversity of endite forms, with tooth-like endites present in the lacinia of some insect maxillae, long needle-like endites in the mosquito proboscis and the long tube-like proboscis of lepidopterans which is formed from maxillary galea endites.

The mandibular gnathal edge of insects (and other mandibulate arthropods) is likely to be derived from endites but the number of endites that make up the mandibular biting edge and the precise manner by which this structure evolved is not known. The principal debate concerns the precise number of endites that make up the mandibular gnathal edge which has generally been interpreted as being comprised of two endites, which develop into the incisor and molar process [2-6]. An alternative view is that the mandibular gnathal edge has evolved from the proximal-most endite (indicated with a star in Figure 1A) on a primitive biramous limb [7,8] meaning that both the incisor and molar processes derive from a single endite (see Figure 1B). Less conventionally, it has been suggested that the mandible gnathal edge is not derived from endites at all, consisting instead of a differentiated substructure not homologous to true endites [9].

The developing embryonic mandibular appendage of insects is a relatively undifferentiated lobe-like structure with few morphological landmarks. Two structural features that are present are the inner and outer lobes, as revealed by SEM studies of embryonic mandibular appendages of various hexapods [5,6,10]. A similar morphology is observed in myriapods, for example, on the embryonic mandible of the millipede *Glomeris marginata*[11]. The inner and outer lobes are generally interpreted as representing the developing molar and incisor processes of the mandible, respectively, although this has not been shown by gene expression or gene function.

From a study of the external morphology of the jumping bristletail (*Pedetontus unimaculatus* Machida), Machida hypothesised that the inner and outer lobe are derived from two separate endites [5]. Machida also hypothesised that the two endites of the maxillary appendage (the lacinia and galea) are serially homologous to the incisor and molar processes, respectively, as they develop in a similar position on their respective appendages (see Figure 1C).

### Expression of genes in the endites

In order to investigate whether the inner and outer lobes are derived from separate endites and whether these later form the incisor and molar processes we compared endite development and expression of a number of genes in the mandibular and maxillary appendages of the red flour beetle *Tribolium castaneum*.

To show whether the mandible has one or two endites and whether the incisor and molar could be homologous to the maxillary lacinia and galea (compare Figure 1B with C) we needed to demonstrate precisely where the incisor and molar develop in the mandibular limb bud. We also needed to investigate endite development in the mandible, in terms of the number of endites and the positions of the developing endites.

Several genes have been shown to be expressed in developing endites of *Tribolium* including the *Tribolium* orthologs of the *Drosophila* genes *paired (Tc-prd)*[12,13], *dachshund (Tc-dac)*[11,14-16] and *Distal-less* (*Tc-Dll*) [17]. We studied the expression of these genes as well as the *Tribolium* homolog of *wingless* (*Tc-wg)* in the developing endites of embryos by *in situ* hybridisation. We followed the expression of these genes into late embryonic development and related the expression patterns of these genes to morphological features of the developing mandible. The morphology of the inner and outer lobes of the mandible was also studied by scanning electron microscopy (SEM). The *pars incisiva* (incisor process) and *pars molaris* (molar process) were examined by light microscopy of cuticle preparations from first instar larvae.

## Methods

### *Tribolium castaneum* culture

Wild-type *Tribolium castaneum* (San Bernardino strain) were kindly provided by Gregor Bucher (Department of Developmental Biology, Georg-August-University Göttingen, Göttingen, Germany) and raised at 32°C in organic wholemeal flour supplemented with 5% brewer’s yeast.

### Cloning of *Tribolium* orthologs

In order to synthesise antisense labelled RNA probes to detect gene expression by *in situ* hybridisation, partial cDNA sequences of *Tc-prd* and *Tc-wg* were identified in the *Tribolium* genome sequence [18] using a BLAST search with *Drosophila* orthologs as the query. Identified sequences were amplified and cloned from mixed stage *Tribolium* cDNA.

*Tc-prd, Tc-Dll, Tc-hth* and *Tc-ser* were amplified from cDNA by PCR amplification using the following primers: *Tc-Dll* (fw: 5’-CAGCAGGTGCTCAATGTGTT-3’ and rv: 5’-ATTAAACAGCTGGCCACACC-3’), *Tc-prd* (fw: 5’-ATGCACAGACATTGCTTTGG-3’ and rv: 5’-GGATCGTCACAGTGTTGGTG-3’), *Tc-hth* (fw: 5’-AGCCGTTTTCTCCAAACAGA-3’ and rv: 5’-GGATAGTGCGCGTACTGGTT-3’), and *Tc-ser* (fw: 5’- AAGGCAACGTTTGCCAATTCGG-3’ and rv: 5’-TCCCATGTGCAACTTCCTGGAGAT-3’, fw: 5’-TCCTTCTGCTACTCAACCTGCTAC-3’ and rv: 5’-GGGGACATTCGCACTTGAACAT-3’, fw: 5’- ATTTGGTGCGGTCTGGGAAACT -3’ and rv: 5’-TCGGGGTTTTGCGCTTTGTAGA-3’). Three sections of *Tc-ser* were amplified and cloned to provide more than 2.5 kb of gene specific sequence and to increase the strength of signal of gene expression in whole-mount *in situ* hybridisation experiments.

Accession numbers are as follows: *Tc-Dll* (GenBank: NM_001039439), *Tc-prd* (GenBank: NM_001077622), *Tc-hth* (GenBank: NM_001039400), and *Tc-ser* (GenBank: XM_964393). A plasmid clone of the *Tribolium* homolog of *dachshund, Tc-dac* (Genbank: XM_964678.2, amplified with primers: fw:5’-CCNGTNGTNTGYAAYGTNGARCARGT-3’ and rv:5’-CKNGCRTTRTCNGCNGCNACYTT-3’) was provided by Dr. Nikola-Michael Prpic-Schäper (Department of Developmental Biology, Georg-August-University Göttingen, Göttingen, Germany).

A plasmid clone of a *Tribolium* homolog of *wingless, Tc-wg* (GenBank: NM_001114350.1, amplified with primers: fw:5’-GGATGCAGGGAAACTGCCTTC and rv: 5’-AACGCAAGTATGTATGGTTCT-3’) was provided by Dr. Andrew Economou (Department of Craniofacial Development, King’s College London, UK).

### Cuticle preparation

Cuticles from first instar larvae were prepared in Hoyer’s medium and lactic acid as previously described [19]. The cuticle preparations were visualised by fluorescence microscopy with an excitation frequency of 488nm.

### Whole mount *in situ* hybridisation

*Tribolium* embryos were fixed in 9% formaldehyde from 24 to 72 h after egglay. Single stainings (nitro blue tetrazolium/5-bromo-4-chloro-3-indolyl phosphate NBT/BCIP) and double stainings (NBT/BCIP and FastRed) were performed as previously described [20]. Some modifications, such as the frequency and duration of washes, were incorporated from alternative *in situ* hybridisation protocols [21].

Stained embryos were dissected from the yolk and mounted in glycerol. Mandibular and maxillary appendages were dissected with sharpened tungsten wire. Embryos and dissected gnathal appendages were visualised using differential interference contrast (DIC) microscopy with an Imager M1 microscope (Carl Zeiss Ltd., Cambridge, UK). Images were taken with Axiocam HRC (Carl Zeiss Ltd., Cambridge, UK) and processed using Axiovision product suite software release 4.8.2. (Carl Zeiss Ltd., Cambridge, UK). Images were processed with GIMP (release 2.6.10) [22].

### Scanning electron microscopy

Embryos were fixed as described for the whole mount *in situ* hybridisation protocol. Fixed embryos were rinsed in ethanol and immersed in hexamethyldisiloxane (HMDS), air dried and sputter coated with gold. Images were taken in a JEOL JSM-5410LV scanning microscope (JEOL Ltd., Tokyo, Japan) at a magnification of 100- to 350-fold and processed with DigitalMicrograph (Gatan Inc., Pleasanton, CA, USA).

## Results and discussion

### Scanning electron micrographs of the inner and outer lobe of the mandible in *Tribolium* embryos

**Figure 2:**
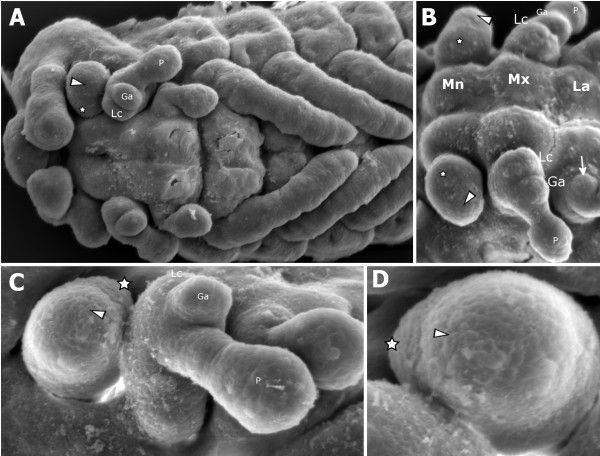
**Scanning electron micrographs (SEMs) of developing gnathal appendages of *Tribolium *embryos showing the inner and outer lobes.** All views are ventral with anterior to the left unless otherwise indicated. The inner lobe is indicated with a star. The outer lobe is indicated with an arrowhead. The labial endite is indicated with an arrow in B. (**A**) Embryo at germ band retracting stage. Endites are visible on the maxillary appendage (Lc - Lacinia and Ga- Galea). The labial appendages have not yet fused at the ventral midline. The inner and outer lobes of the mandible are faintly distinguishable. (**B, C**) Lateral view of mandibular, maxillary and labial appendages of an embryo at a similar stage to A. (**D**) Close-up of mandibular limb bud with clearly distinguishable inner and outer lobes present. Anterior is bottom, lateral is to the right. Mandibular (Mn), maxillary (Mx) and labial (La) segments are indicated, as are the lacinia (Lc), galea (Ga), and maxillary palp (P).

Scanning electron micrographs (SEMs) were taken of *Tribolium* embryos to relate the embryonic inner and outer mandibular lobes to the expression of the various genes of interest (Figure 2) using high resolution images. The developing mandible has two lobes perpendicular to the limb’s proximal-distal (PD) axis. The proximal lobe (the inner lobe - star in Figure 2), relates to the molar process and the distal lobe, (the outer lobe - arrowhead in Figure 2), relates to the incisor process and these are distinguishable in the developing mandible of germ band extending embryos (see Figure 2). We base this correlation on the expression of *Tc-prd,* which marks the developing inner and outer lobes during embryogenesis (see below).

The inner and outer lobes resemble the maxillary endites, the lacinia and galea, and are in a comparable position along the PD axis. However, the inner and outer mandibular lobes are larger and less defined than the maxillary endites (Figure 2A to C).

SEM studies in other insects have demonstrated that the inner and outer lobes are present in the cricket *Gryllus assimilis*[10], the sawfly *Athalia rosae*[6] and the jumping bristletail *Pedetontus unimaculatus*[5]. A study into the expression of PD domain genes in the gnathal appendages of the millipede *Glomeris marginata* similarly shows a mandible with an inner and an outer lobe [11].

These lobes are often interpreted as endites, but aside from similarities of morphology and position of development, there is little other evidence to support this interpretation. We wanted to examine the expression of marker genes for the development of endites to see if we could determine if the inner and outer lobes can reasonably be interpreted as separate endites.

### *Tc-prd* marks the developing endites in the embryonic gnathal appendages

**Figure 3:**
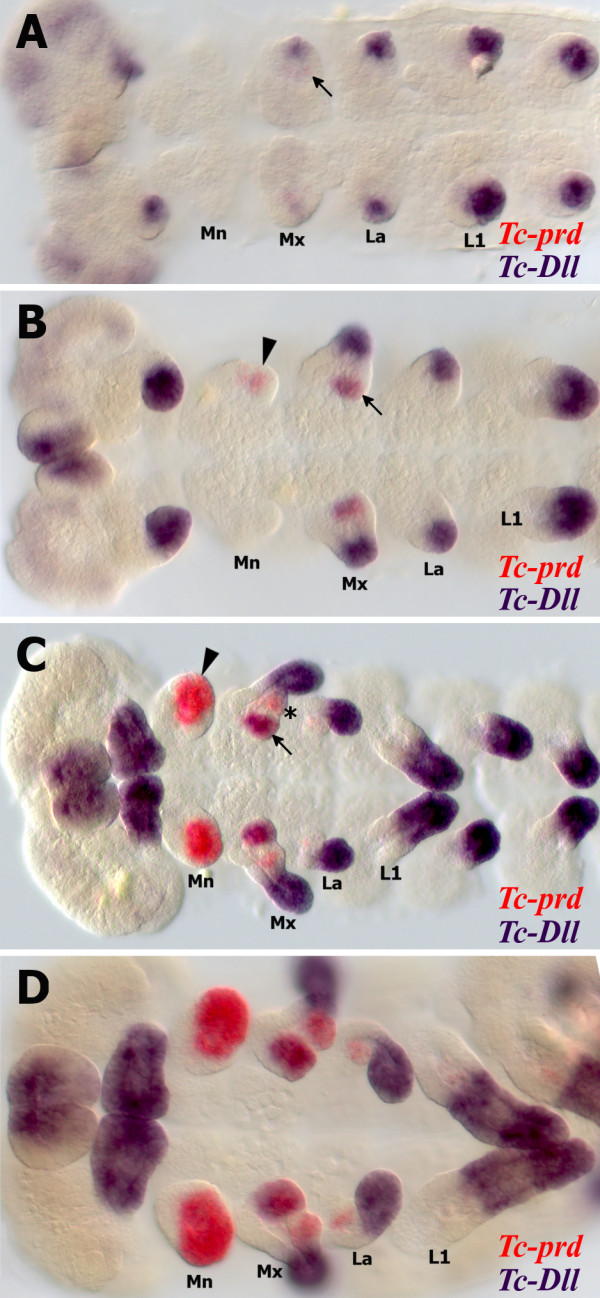
***Tc-prd *marks the developing endites in the embryonic gnathal appendages*.*** All views are ventral with anterior to the left unless otherwise indicated. (**A**) Germ band extending stage embryo after formation of the limb buds, which are marked by *Tc-Dll* expression. Faint *Tc-prd* expression is visible in the maxilla (arrow). **(B**) Later germ band extending stage embryo. *Tc-prd* expression is now visible in the mandible (arrowhead). Expression of *Tc-Dll* and *Tc-prd* is present the maxillary lacinia endite (arrow). (**C**) Fully germ band extended stage embryo. The endites of the mandible, maxilla and labial appendages are marked by *Tc-prd* expression. *Tc-Dll* expression is present in the lacinia endite lobe (arrow) but absent from the galea (asterisk). The mandible has one domain of *Tc-prd* expression which is significantly larger than either of the maxilla endite expression domains. (**D**) Germ band retracting stage embryo, Expression of *Tc-prd* and *Tc-Dll* is maintained in the same domains as in C.

**Figure 4:**
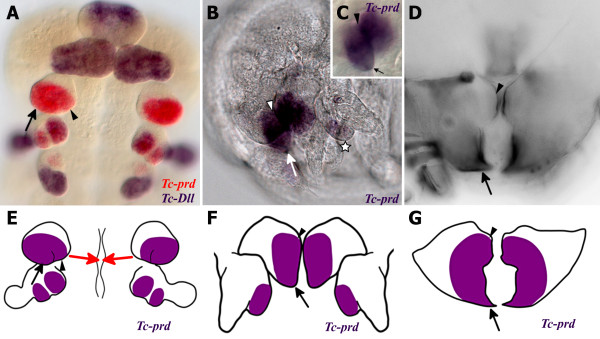
**Expression of *Tc-prd *in the embryonic mandibular endite relates the inner and outer lobe to the incisor and molar processes.** (**A**-**G**) The outer lobe/incisor process are indicated with arrows. The inner lobe/molar process are indicated with arrowheads. (**A**) Ventral view of *Tc-prd* (red) and *Tc-Dll* (blue) expression in the developing embryonic mandible of a germ band retracting stage embryo. Anterior is up. (**B**) Expression of *Tc-prd* in the mandible endite of a very late stage embryo prior to formation of the cuticle. Anterior is left, dorsal is up. *Tc-prd* is also expressed in the maxillary ventral branch consisting of fused lacinia and galea (star). (**C**) Close-up of the right mandible shown in B. *Tc-prd e*xpression is visible in the pointed distal tip, the presumptive incisor (arrow) and molar (arrowhead) process of the mandibular endite. (**D**) Mandibles of a first instar larva visualised by fluorescence microscopy. The mandible is unsegmented, with the gnathal edge consisting of an incisor process and molar process. (**E**,** F**) Diagrams of *Tc-prd* expression in the developing mandible. The expression of *Tc-prd* is in one domain which encompasses the inner and outer lobes. (**E**) Diagram outlining the shape of both the mandible and maxilla from A with *Tc-prd* expression (purple). The mandibles will move towards the midline, see red arrows. (**F**) Diagram outlining the shape of both the mandible and maxilla from B with *Tc-prd* expression (purple). Mandibles have moved toward the midline. (**G**) Outline of the larval mandible shown in D with hypothetical expression of *Tc-prd* (purple) superimposed based upon the expression of *Tc-prd* in B and C.

In order to show where the endites develop in the gnathal appendages, we studied expression of *Tc-prd*, which, in addition to its role as a pair rule gene, is expressed in the endites of the maxillary and labial appendages during embryogenesis as well as the inner and outer lobes of the mandible [13] soon after formation of the limb buds (Figure 3B) and is continually expressed in the gnathal appendage endites throughout embryogenesis. Any changes that occur to the endites during embryogenesis are associated with a corresponding change in *Tc-prd* expression. When the lacinia and galea endites fuse to form the ventral branch, the two *Tc-prd* expression domains also fuse (see star in Figure 4B). Apparent loss or reduction of the labial endite by late embryogenesis results in loss of *Tc-prd* expression (see Figure 4B).

There are two domains of *Tc-prd* expression in the maxillary appendages that relate to the two endites, which are likely to be the presumptive lacinia and galea (Figure 3C, D). The endite expression domain of *Tc-prd* first appears in the maxilla in the developing lacinia endite (arrow in Figure 3A, B).

*Tc-Dll* is expressed in the telopodites of all limb bud primordia except the mandible (Figure 3C, D). A proximal domain of *Tc-Dll*, distinct from the distal telopodite domain is expressed in the developing maxillary endites. Expression of *Tc-Dll* is first seen in the lacinia, and appears in the galea at a later stage [23]. As *Tc-Dll* is co-expressed with *Tc-prd* in the first proximal domain to appear, it is likely that this is the lacinia endite (arrow in Figure 3A, B). The galea endite forms during a later stage (Figure 3C).

In the mandible we can see a single domain of *Tc-prd* expression (Figure 3C, D) which is significantly larger than either of the two domains found in the maxillary or labial appendages. There is no evidence that *Tc-prd* expression in the mandible is divided into two separate domains. There is one domain of *Tc-prd* expression in the labial appendages (Figure 3D).

Considering the position and timing of expression of *Tc-prd* in the mandibular limb bud compared to the endites of the maxillary and labial appendages, it seems reasonable to conclude that *Tc-prd* expression in the mandible is also an endite-specific expression domain.

### The mandibular inner and outer lobes are the developing molar process and incisor process, respectively

In order to show where the gnathal edge develops on the mandibular limb bud, we studied *Tc-prd* expression through to later stage embryos when the mandible more closely resembles the larval mandible.

In earlier stages of development, *Tc-prd* expression is present throughout the inner and outer lobes (Figure 4A). In very late stages, immediately prior to the formation of the cuticle, the single domain of *Tc-prd* expression covers most of the presumptive gnathal edge including the developing incisor (arrow in Figure 4B, C) and molar (arrowhead in Figure 4B, C) processes.

At this stage, the two maxillary endites have fused to form the ventral branch of the maxilla (star in Figure 4B) and there is only one corresponding domain of *Tc-prd* expression in the maxillae. The ventral branch later develops into the lacinia and galea in adult beetles [24], however the expression of *Tc-prd* is not known at this stage.

The morphology of the embryonic mandibles at this late stage (Figure 4B, C, F) is beginning to resemble the morphology of the larval mandibles (Figure 4D, G). The outer lobe has elongated to form the distal point of the incisor process (arrow in Figure 4C). Both mandibles have moved to the midline in front of the mouth (see red arrows in Figure 4E and compare to Figure 4F) and the inner and outer lobes are now adjacent to each other as they are in the first instar larvae (see Figure 4D).

Assuming that there is no significant change in *Tc-prd* expression during later embryogenesis, the expression of *Tc-prd* in the developing incisor and molar processes of a late stage *Tribolium* embryo is evidence that the inner lobe relates to the molar process and the outer lobe relates to the incisor process. In Figure 4G we show where this domain of *Tc-prd* expression most likely relates to in the larval mandible.

### The expression patterns of *Tc-prd, Tc-wg* and *Tc-dac* in the mandible and maxilla suggest the mandible may be composed of one endite

Having demonstrated that the inner and outer lobes are likely to relate to the molar and incisor processes, respectively, we wanted to determine whether the inner and outer lobes develop from separate endites or from a single endite. There is a one-to-one correspondence of *Tc-prd* expression and the development of endites (or lobes such as the ventral branch) through ontogeny. The single large domain of mandibular *Tc-prd* expression could indicate that both molar and incisor derive from a single endite. An alternative interpretation, however, is that this single domain of *Tc-prd* corresponds to the fusion of two endites. In order to try to differentiate between these two possibilities we compared the expression of *Tc-prd* with additional genes that have characteristic expression domains in the endites of the gnathal appendages.

### *Tc-wg* expression in the mandibular and maxillary appendages

Previous studies in *Tribolium* and *Schistocerca* of the expression of homologs of *wingless* (*wg*), a gene with important roles in segmentation and limb development, showed that *wg*, while expressed in a ventral stripe in insect limbs, specifically fades at the locations of the forming maxillary and labial endites [23]. A similar situation seems to be apparent in the millipede *Glomeris*[25]. In order to help determine the endite composition of the mandible we examined the expression of *Tc-wg* in the mandible.

**Figure 5:**
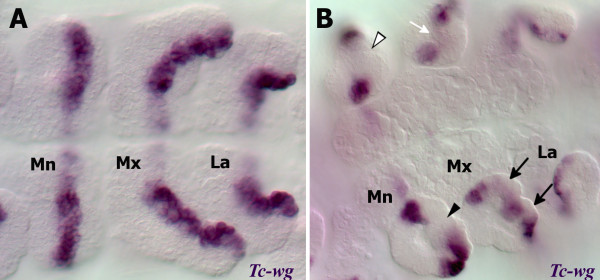
**The gaps in the expression pattern Tc-wg in the mandible and maxillary endites suggest the mandible is composed of one endite.** All views are ventral with anterior to the left. Gene expression was detected by *in situ* hybridisation. (**A**) Fully extended germ band stage embryo. *Tc-wg* is expressed in parasegmental stripes and in the medial region in the ventral ectoderm of each developing appendage. Expression is continuous throughout the medial part of each appendage including the developing endites. (**B**) Germ band retracting stage embryo. There is a gap of *Tc-wg* expression in the mandibular endite (black arrowhead) and two gaps in the maxillary endites (black arrows). A white arrowhead marks the boundary between the inner and outer lobes which is lacking *Tc-wg* expression. A white arrow shows where there is a lack of ectodermal expression in the developing maxillary lacinia. Mandibular (Mn), maxillary (Mx) and labial (La) segments are indicated.

As in other arthropods, *Tc-wg* is expressed in a stripe that runs through the middle of the ventral ectoderm of all appendages (Figure 5A). In the maxilla, *Tc-wg* expression retracts from both of the two endites to form two gaps in *Tc-wg* expression (see arrows in Figure 5B). There is no expression in the developing lacinia endite (see white arrow in Figure 5B).

In the mandible, only one gap in *Tc-wg* expression develops (arrowhead in Figure 5B). The groove or boundary between the inner and outer lobes of the mandible does not have *Tc-wg* expression (see white arrowhead in Figure 5B). By contrast, the groove between the maxillary endites shows *Tc-wg* expression. This single gap in the ventral domain of *Tc-wg* expression in the mandible would therefore support the view that there is only a single endite present.

### *Tc-dac* expression in the mandibular and maxillary endites

Homologs of *dachshund (dac*) are expressed in the distal part of developing endites of mandibulates. In the cricket *Gryllus* for example*, Gm-dac* is expressed in the distal half of the maxillary and labial endites [15]. In the crustaceans *Triops* and *Thamnocephalus,* the homolog of *dac* is expressed in the distal half of each of the five endites present on the phyllopodous limbs [16].

The characteristic expression pattern of *dac* homologs in endites suggests that *dac* may be used as a marker of endite development in the embryonic mandible. *dac* homologs are strongly expressed in distal part of the mandible in the cricket *Gryllus*[15], the crustacean *Porcellio*[26] and the millipede *Glomeris*[11]. We were interested in the expression of *Tc-dac* and whether it could inform us of the endite composition of the mandible appendage.

**Figure 6:**
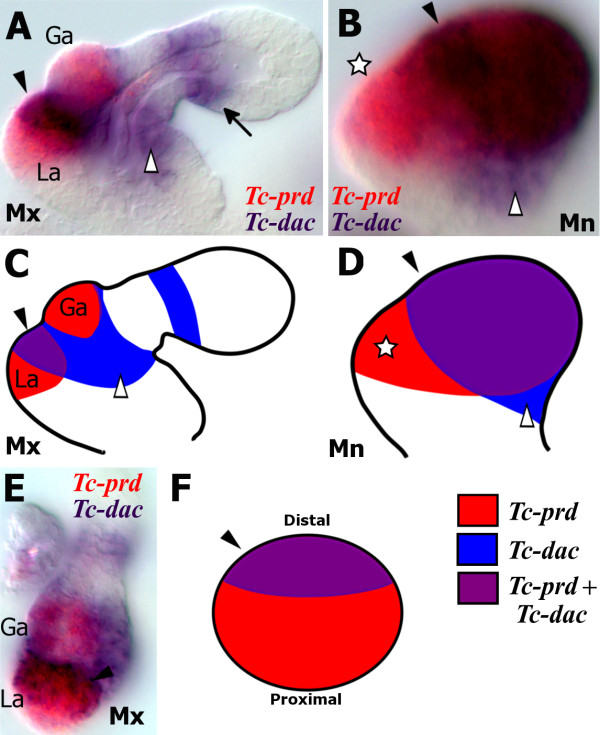
**Comparison of the expression patterns of Tc-prd and *Tc-dac *in the mandible and maxilla suggest the mandible is composed of one endite.** All views lateral is to the right and distal is top unless otherwise indicated. (**A**) *Tc-dac* (blue) and *Tc-prd* (red) expression in the dissected maxilla of a germ band retracting embryo. There are two domains of *Tc-dac* expression, a proximal domain (white arrowhead) and a distal domain (arrow). *Tc-dac* expression overlaps with *Tc-prd* in the distal half of the developing lacinia endite (black arrowhead). *Tc-dac* expression is lacking from the proximal half of this endite and faint in the galea endite. (**B**) Expression of *Tc-dac* (blue) and *Tc-prd* (red) in a dissected mandible of a germ band retracting embryo. *Tc-dac* is co-expressed with *Tc-prd* in the outer lobe (arrowhead). *Tc-dac* expression is lacking in the inner lobe (star). The proximal domain of *Tc-dac* which is not co-expressed with *Tc-prd* is indicated (white arrowhead). (**C**) Diagram of the dissected maxilla shown in A. (**D**) Diagram of dissected mandible shown in B. (**E**) Ventral view of dissected maxilla, distal is top. (**F**) Simplified representation of the endite expression of *Tc-prd* (red) and *Tc-dac* overlap (purple) in an endite. Compare with the developing lacinia shown in E. Distal to the top.

There are two domains of *Tc-dac* expression in post-antennal appendages of *Tribolium*, a proximal domain and a distal domain [14]. The proximal and distal domains of *Tc-dac* in the maxilla are shown in Figure 6A.

In the maxilla, the proximal domain of *Tc-dac* includes expression in the distal protopodite, which lacks *Tc-prd* expression (see white arrowhead in Figure 6A, C), and the endites. In the endites that are marked by *Tc-prd* expression, *Tc-dac* is expressed in the distal half of the lacinia endite (arrowhead in Figure 6A). Unlike in the cricket *Gryllus, Tc-dac* expression is faint in the galea endite of the maxilla. Expression of *Tc-dac* was too faint to determine endite specific expression in the labial endite.

In the embryonic mandible, *Tc-dac* is expressed in the distal part of the mandible (as in *Gryllus* and *Porcellio*) which includes co-expression in the outer lobe with *Tc-prd* (black arrowhead in Figure 6B) and expression in the lateral protopodite which lacks *Tc-prd* expression (see white arrowhead in Figure 6B, D). *Tc-dac* is not expressed in the inner lobe (white star in Figure 6B). The mandibular expression of *Tc-dac* and *Tc-prd* resembles the expression of these genes in the developing lacinial maxillary endite (see Figure 6F).

The lack of a distal endite domain in the galea endite discredits the use of *Tc-dac* as an endite marker to some extent. However the expression pattern of *Tc-dac* in the lacinia endite is similar to that of *Gb-dac* in the lacinia and galea endites of the cricket *Gryllus* which does have a separate lacinia and galea in the first instar larval stage unlike *Tribolium*[15]. The presence of these two endites, the lacinia and galea, in the maxilla is ancestral for the Hexapoda [1]. For these reasons we suggest that using *Tc-dac* as a marker for the distal part of the mandibular and lacinia endites is valid. Expression of *Tc-dac* in the outer lobe and lack of expression of *Tc-dac* in the inner lobe suggests, therefore, that the outer lobe is the distal part of a single mandibular endite.

**Figure 7:**
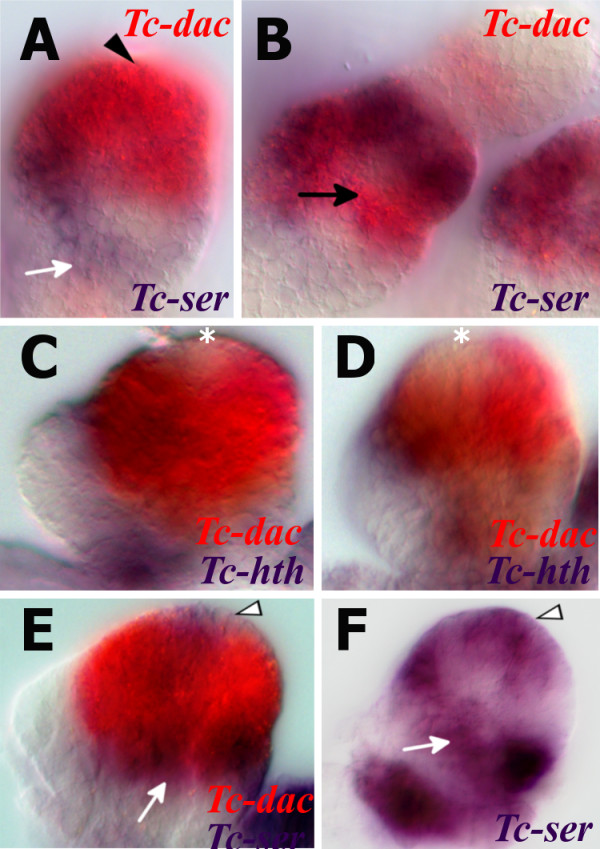
**A circular gap of *Tc-dac *expression in the outer lobe of the mandible is filled by a ‘cap’ of *Tc-ser *expression.** All views are ventral with anterior to the left unless otherwise indicated. Gene expression was detected by *in situ* hybridization. (**A**) *Tc-dac* (red) and *Tc-ser* (blue) expression in the mandible of a germ band extending embryo. Ventral view with distal at top. *Tc-dac* is expressed continuously in the outer lobe of the mandible. A ring of *Tc-ser* (white arrow) which we interpret as the distal boundary of the subcoxa is indicated (Coulcher, manuscript in preparation). (**B**) *Tc-dac* (red) and *Tc-ser* (blue) expression in a maxilla of the same embryo as shown in A. Ventral view with distal top. *Tc-dac* is expressed in the distal part of the developing lacinia endite. The lacinia and galea endite lobes have formed. (**C**) *Tc-dac* (red) and *Tc-hth* (blue) expression in a germ band extended embryo. A circular region of *Tc-dac* expression is missing in the outer lobe (asterisk). *Tc-hth* is expressed in the very distal part of the inner lobe, and is lacking entirely from the outer lobe. (**D**) *Tc-dac* (red) and *Tc-hth* (blue) expression in a germ band extended embryo. The circular gap in expression (asterisk) widens as development progresses. (**E**) *Tc-dac* (red) and *Tc-ser* (blue) expression in a dissected mandible. A ‘cap’ of *Tc-ser* expression is expressed within the circular gap of *Tc-dac* expression in the outer lobe (white arrowhead). The subcoxal ring of *Tc-ser* expression is indicated with a white arrow. **(F**) *Tc-ser* expression in a dissected mandible. Lateral view with distal top. A ‘cap’ of *Tc-ser* expression is present at the distal-most point of the outer lobe (white arrowhead). The subcoxal ring of *Tc-ser* expression is indicated with a white arrow.

Interestingly, as the mandibular bud develops, *Tc-dac* expression is lost from a circular region in the distal-most part of the outer lobe of the developing mandible. *Tc-dac* is expressed continuously in the outer lobe of the mandible during earlier stages when the inner and outer lobes have begun to form (Figure 7A). In the maxillae of the same embryo, the lacinia and galea have already formed (Figure 7B). The circular gap of *Tc-dac* expression in the outer lobe of the mandible does not appear till later (asterisk in Figure 7C, D), after the endites have formed.

Study of the expression of the *Tribolium* homolog of *serrate* (*Tc-ser*) shows that there is a cap of *Tc-ser* expression (see arrowhead in Figure 7F) that appears mutually exclusive with the expression of *Tc-dac* in the outer lobe (see arrowhead in Figure 7E). Considering the position of this *Tc-ser* cap domain at the distal most point of the outer lobe on the mandibular limb bud, we propose that this is the presumptive incisor process.

### *Tc-dac* expression suggest that the mandible has one endite

**Figure 8:**
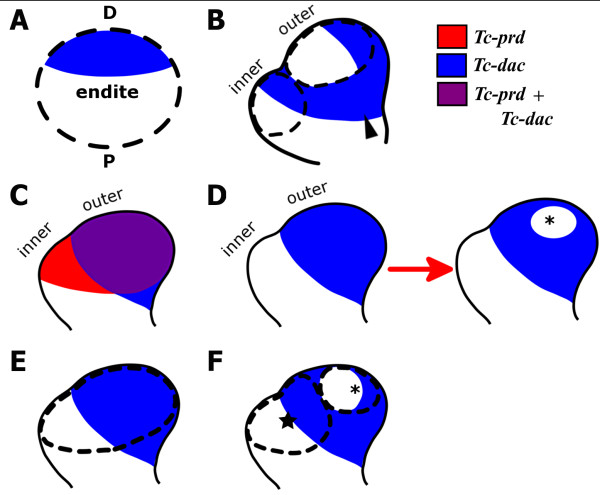
**Different hypotheses of the endite composition of the mandible based upon expression of *Tc-dac.*** All views are medial to the left with distal top. Developing endites are represented by a dotted circle. *Tc-dac* expression is blue. (**A**) Schematic representation of *Tc-dac* expression in the distal part of an endite. (**B**) Hypothetic *Tc-dac* expression if the inner lobe and outer lobe are separate endites. *Tc-dac* is expressed in the distal part of the two endites. Non-endite expression of the proximal domain of *Tc-dac* also includes expression in the distal part of the protopodite (arrowhead). (**C**) Diagram of the actual expression of *Tc-dac* (shown in blue) and *Tc-prd* (shown in red)*. C*o-expression of *Tc-prd* and *Tc-dac* is purple. *Tc-prd* is expressed in the inner and outer lobes. (D) Diagram of the actual expression of *Tc-dac*. *Tc-dac* is expressed in the outer lobe, and is lacking in the inner lobe. A circular gap in *Tc-dac* expression (asterisk) later appears in the outer lobe. (**E**) The hypothesis presented in this study, where there is one mandibular endite which includes both the inner and outer lobe. *Tc-dac* is expressed in the outer lobe, the distal part of the endite. (**F**) An alternative hypothesis based on *Tc-dac* expression where the mandible has two endites (indicated with a star and asterisk). There is no expression of *Tc-dac* in the inner lobe. Therefore the two endites do not correlate to the inner and outer lobe precisely. Rather, the proximal endite (star) includes the inner lobe and the proximal part of the outer lobe. The distal endite (asterisk) includes part of the outer lobe.

As noted above, homologs of *dac* have characteristic expression pattern in the distal part of endites of mandibulate arthropods (Figure 8A). The expression of *dac* homologs in the distal part of the mandible (the outer lobe) therefore appears to be conserved in mandibulate arthropods. Using *Tc-dac* as a marker for endite development, we would expect there to be *Tc-dac* expression in the distal part of each lobe if both the inner and outer lobe are two separate endites (Figure 8B).

What we have shown however, is that *Tc-dac* is expressed in the outer lobe but is not expressed in the inner lobe of the mandible (Figure 8C,D). This suggests that the outer lobe of the mandible could represent the distal part of a singular mandibular endite (Figure 8E).

It could be argued that the circular gap of *Tc-dac* expression in the outer lobe (asterisk in Figure 8D) of mandible limb bud that appears later represents a second endite. However, *Tc-dac* is not expressed in the inner lobe (particularly the distal part of the inner lobe) which argues against the hypothesis that the inner lobe itself is a separate endite. The two endites, if they exist, would therefore both develop on the outer lobe (Figure 8F). However, there is no obvious endite structure (like a lobe) that could relate to the supposed second endite in the distal part of the outer lobe and we have shown that it is the inner and outer lobes that develop into the incisor and molar processes. This gap of *Tc-dac* expression appears later during embryogenesis after the endites of other appendages have already formed.

We suggest that the gap in *Tc-dac* expression is not likely to represent the proximal part of an additional developing endite on the outer lobe, but is more parsimoniously interpreted as representing an expression pattern that is unique to mandibular development, possibly relating to the later development of the incisor process.

### Alternative interpretations of the expression data

Although our data are suggestive of the existence of a single mandibular endite, there are alternative explanations that we have not been able to rule out.

Firstly, there are likely to be differences between the endite (or endites) of the mandible and the maxillary endites, which reflect the morphological differences between the two appendage types. These differences could result in differences in gene expression and function. One such difference could be that the inner and outer lobe are two endites that have ‘fused’ to some extent, such that *Tc-prd* expression has one domain for example.

Furthermore, as the mandible is a derived structure, it seems likely that there will be novel aspects to its development. For example, regarding the expression of *dac* homologs in the distal part of the mandible, this could represent part of a novel mechanism by which the mandible is patterned and therefore not directly comparable to endites in other appendages. *dac* homologs may have roles in the formation of the outer lobe that is particular to the mandible.

Finally, and most importantly, explanations of how endites are patterned are currently lacking. Preliminary functional investigations into endite patterning genes have been performed in the dung beetle *Onthophagus*[27] and *Tribolium*[28]. Comparing the endite-patterning function of genes between diverse arthropod taxa may be able to answer questions of endite homology in arthropod appendages and the endite composition of the mandible. With reference to our study, the possible endite patterning functions of either *Tc-prd, Tc-dac* or *Tc-wg* are not known. Of particular interest is the function of these genes in *Tribolium* and the possible endite patterning function of *Tc-prd* homologs*,* the Pax group III genes [29-31] and *dac* homologs across Arthropoda*.*

### Serial homology of the mandibular endite to those of the maxilla

This study has relied upon gene expression markers to conclude that the mandible has one endite from which both incisor and molar processes develop. This conclusion would contradict Machida’s hypothesis of serial homology of the incisor and molar process to the galea and lacinia of the maxilla. Machida’s hypothesis has previously been questioned on morphological grounds. Boxshall, for example, rejects Machida’s hypothesis because the endites are present on separate segments in the primitive hexapod maxilla, whereas there is no evidence for a segmental division of the mandibular gnathobase [1].

There still remains the question of serial homology of the mandibular endite, whether it is homologous to either of the maxillary endites, the lacinia or galea.

Consideration of fossil evidence, may also be illuminating: the proximal-most endite present on the primitive biramous limb of Cambrian arthropods such as *Martinssonia* and *Henningsmoenicaris* as well as many others [7,8,32] has been proposed as a precursor to the mandible gnathal edge which is also the most proximal endite. If there is a serially homologous endite to the mandibular endite, therefore, the most reasonable candidate within the Hexapoda would be the proximal-most endite of the maxilla, that is the lacinia,.

## Conclusion

The gene *Tc-prd*, in addition to its pair-rule function in segmentation, has additional expression domains in the inner and outer lobes of the mandible and the developing endites of the maxillary and labial appendages. Following expression of the single mandibular domain of *Tc-prd* into late stage *Tribolium* embryos shows that the inner and outer lobes make up the embryonic mandibular endite and develop into the future incisor and molar processes.

Comparing expression of the Proximal-Distal appendage domain gene *Tc-dac* and markers for endite development (*Tc-prd* and *Tc-wg*) suggests that the inner and outer lobes may be derived from a single endite. As there is only one mandibular endite that divides into two lobes that develop into the molar and incisor processes, we conclude that the mandibular endite has evolved from a single typical lobe-like endite to form an endite consisting of two parts. The mandible endite would have expanded proximally to form the molar process (inner lobe) and distally to form the incisor process (outer lobe). The inner lobe is derived from the proximal half of the endite which is lacking *Tc-dac* expression and is marked by *Tc-prd* expression, and the outer lobe is derived from the distal half of the endite (marked by *Tc-dac* and *Tc-prd* expression).

Our tentative conclusion, therefore, is that Machida’s hypothesis that the mandible incisor and molar processes are derived from two separate endites homologous to the maxillary galea and lacinia is incorrect and that the gnathal edge of the *Tribolium* mandible is more likely to have derived from a single endite which may be serially homologous to the proximal-most endite of the maxilla: the lacinia.

## Abbreviations

La: Labial appendage; L1: first thoracic leg; Mn: Mandible; Mx: Maxilla; PD: Proximal-distal; *Tc-Dll*: the *Tribolium* ortholog of *Distal-less (Dll)*; *Tc-prd*: the *Tribolium* ortholog of *paired (prd)*; *Tc-dac*: the *Tribolium* ortholog of *Dachshund*; *Tc-wg*: the *Tribolium* ortholog of *wingless (wg)*; *Tc-hth*: the *Tribolium* ortholog of *homothorax (hth)*; *Tc-ser*: the *Tribolium* ortholog of *serrate (ser)*.

## Competing interests

The authors declare that they have no competing interests.

## Authors' contributions

JFC and MJT conceived and designed the study. JFC collected the data and analysed the results. JFC and MJT drafted the manuscript and approved the final manuscript for submission. Both authors read and approved the final manuscript.
